# Versatile and Low-Cost Fabrication of Modular Lock-and-Key Microfluidics for Integrated Connector Mixer Using a Stereolithography 3D Printing

**DOI:** 10.3390/mi13081197

**Published:** 2022-07-28

**Authors:** Isa Anshori, Vincent Lukito, Rafita Adhawiyah, Delpita Putri, Suksmandhira Harimurti, Tati Latifah Erawati Rajab, Arfat Pradana, Mohammad Akbar, Mas Rizky Anggun Adipurna Syamsunarno, Murni Handayani, Agnes Purwidyantri, Briliant Adhi Prabowo

**Affiliations:** 1 Biomedical Engineering Department, School of Electrical Engineering and Informatics, Bandung Institute of Technology, Bandung 40132, Indonesia; lukitov@gmail.com (V.L.); rafitaadhawiyah@gmail.com (R.A.); delpita1putri@gmail.com (D.P.); sukman@staff.itb.ac.id (S.H.); tati@stei.itb.ac.id (T.L.E.R.); 2 Research Center for Nanosciences and Nanotechnology (RCNN), Bandung Institute of Technology, Bandung 40132, Indonesia; arfatpradana@gmail.com; 3 Department of Cardiology and Vascular Medicine, Faculty of Medicine, Universitas Padjadjaran and Dr. Hasan Sadikin General Hospital, Bandung 40161, Indonesia; m.r.akbar@unpad.ac.id; 4 Department of Biomedical Sciences, Faculty of Medicine, Universitas Padjadjaran, Bandung 45363, Indonesia; rizky@unpad.ac.id; 5 National Research and Innovation Agency (BRIN), Tangerang Selatan 15314, Indonesia; murni.handayani@brin.go.id (M.H.); agnes.purwidyantri@inl.int (A.P.); 6 International Iberian Nanotechnology Laboratory (INL), 4715-330 Braga, Portugal

**Keywords:** 3D printing, microfluidics, two lock-and-key modular, submillimeter scale, additive manufacturing, gradual design, printing orientation, dimension limit, low cost, hollow structure

## Abstract

We present a low-cost and simple method to fabricate a novel lock-and-key mixer microfluidics using an economic stereolithography (SLA) three-dimensional (3D) printer, which costs less than USD 400 for the investment. The proposed study is promising for a high throughput fabrication module, typically limited by conventional microfluidics fabrications, such as photolithography and polymer-casting methods. We demonstrate the novel modular lock-and-key mixer for the connector and its chamber modules with optimized parameters, such as exposure condition and printing orientation. In addition, the optimization of post-processing was performed to investigate the reliability of the fabricated hollow structures, which are fundamental to creating a fluidic channel or chamber. We found out that by using an inexpensive 3D printer, the fabricated resolution can be pushed down to 850 µm and 550 µm size for squared- and circled-shapes, respectively, by the gradual hollow structure, applying vertical printing orientation. These strategies opened up the possibility of developing straightforward microfluidics platforms that could replace conventional microfluidics mold fabrication methods, such as photolithography and milling, which are costly and time consuming. Considerably cheap commercial resin and its tiny volume employed for a single printing procedure significantly cut down the estimated fabrication cost to less than 50 cents USD/module. The simulation study unravels the prominent properties of the fabricated devices for biological fluid mixers, such as PBS, urine and plasma blood. This study is eminently prospective toward microfluidics application in clinical biosensing, where disposable, low-cost, high-throughput, and reproducible chips are highly required.

## 1. Introduction

Microfluidics devices sparked a potential light in the point-of-care diagnostics revolution. These systems enable integration with a myriad of analytical detection strategies to establish rapid analysis and less-laborious operations, using minute volumes of samples and reagents [1,2,3]. Additionally, these devices offer versatility, targeting a broad range of applications, such as sample preparation, sample delivery, sample waste, sample handling, and modeling organ-on-a-chip [4,5,6,7,8,9]. A prominent trajectory in microfluidics is the micro-total analysis system (µ-TAS), which holds the ultimate premises of comprehensive integration of a sensing element and sample handling system, including manipulations of small volumes in the microchannel and data processing module [10,11,12,13].

In microfluidics device production, the conventional fabrication techniques, for instance, soft lithography, remain a major shortcoming due to their long process, the requirement for masking, and operation by trained technicians. Moreover, despite other maskless methods, such as engraving mold using a laser and milling machine, which were reported to simplify the molding fabrication process [14,15,16], the structure resolution is still hard to compete with the photolithography process using the mask. Meanwhile, polymer casting is noticed to be the most critical step for microfluidics fabrication [17,18]. The casting method is the main challenge for the commercialization of microfluidics devices because of its low reproducibility and throughput of yields. These limitations inflict a significant gap between lab prototyping, scale-up production needs, and market standardization [18,19].

Three-dimensional printing is one of the disruptive technologies in the microfluidics field. It speeds up the molding fabrication and makes it feasible for direct microfluidics fabrication without polymer casting, owing to the high availability of the various resin types in the market [20,21,22,23,24]. Among the number of distinctive printing techniques, such as laser sintering, fused filament, and an inkjet-based 3D printer, resin stereolithography emanates as a potential fabrication pathway of 3D printing because of its high resolution, good surface finishing, and considerably low expense per printed device. Stereolithography is a remarkable process of exposing photosensitive monomers layer by layer using a controlled ultraviolet light source to the resin. The master file of the 3D model can be designed by commercial or open-source software and loaded into the 3D printer machine. The curing methods include stereolithography with direct laser writing, digital light processing, continuous liquid interface production (CLIP), and continuous digital light manufacturing (CDLM), which are varied approaches to the mechanism of exposing the resin to define each fabricated layer [25]. The structure is constructed by a slice-per-slice pattern in the X-Y direction, which later moves on to the Z direction for forming subsequent layers until the 3D structure is completed. Curing the deposited liquid resin layer by layer gives an advantage of faster printing and an optimal observational view compared to filament-based 3D printing, which is generally afflicted by visible printing lines whenever each layer is formed. 

There are critical features for functional microfluidics for bio-clinical applications, such as single-use application, biocompatibility, disposability, reliability, cost-effectiveness, modularity, and high reproducibility, especially for cohort studies. In particular, the last three features could be overcome by using 3D-printed devices. For example, high-throughput yields significantly reduce production costs with a reasonable and scalable production time [21,26,27]. Furthermore, the reproducibility of 3D-printed modular microfluidics is more controllable compared to conventional casted microfluidics [26,28]. Nie et al. showed the capillary-driven modular microfluidics inspired by Lego^®^ [27]. The modular microfluidics were assembled from individual functional modules, which provide versatility and flexibility for the reconfiguration and customization of microfluidics devices. 

The straightforward fabrication of complex structures with a resolution under millimeter scale using entry-level and open-source hardware 3D printing could be an alternative for the engineering of small-dimension structures [29]. Several microfluidics application requires hundreds micrometer features [14,30,31], which can be achieved using low cost 3D printing, such as connector, mixer or serpentine channels. Therefore, the simplified structures and fabrication method can be improved for better modularity and disposability features. Several studies presented a commercial connector that separated the mixer microfluidics [30,32,33]. The simplification that the serpentine mixer can be integrated to the lock and key connector is feasible for fabrication using 3D printing.

Currently, details on optimization parameters in printing 3D microfluidics using a low-cost, entry-level 3D printer of less than USD 500 have not yet been investigated, while most of the works related to 3D microfluidics were fabricated using systems higher than USD 5000, which have much better optical components, electronics, software, mechanical systems, and resin chemicals. In addition, most low-cost 3D printers are geared toward fabricating at the millimeter scale and upward. Thus, detailed know-how has not been of major interest to the user. Therefore, an early adopter of the technology for microfluidics studies might be beneficial for thorough guidance in printing optimization using affordable 3D printers to obtain fast prototypes.

This article presents the fabrication of novel integration lock-and-key microfluidics modules for the production of connector and chamber modules. Our proposed structure of an integrated modular key lock connector and serpentine microfluidics, to the best of our knowledge, the first one being produced by low cost 3D printing. These two components would be the pivotal features for versatile microfluidics applications. The zig-zag connector for the mixer of liquid samples was fabricated and optimized to explore the potencies of the low-cost LCD-resin 3D printer. Next, the connector could be integrated into a single functional microfluidics device. We initially investigated the structural design evaluation and printing parameters. The next part entails the detailed strategy for the submillimeter fluid chamber module creation.

## 2. Materials and Methods

### 2.1. Materials

Modular blocks were designed with AutoCAD 2018 (Autodesk^®^Inventor^®^ Fusion, Autodesk Inc., San Rafael, CA, USA). The designs were exported into.stl files and sliced using slicer software ANYCUBIC PhotonSlicer (Anycubic, Shenzhen, China) or CHITUBOX 64 (CHITUBOXTM, Shenzhen, China). The slicing process resulted in a photon file compatible with the digital light processing stereolithographic (DLP-SLA) 3D printer from Anycubic Photon S (Anycubic, Shenzhen, China) used in this study. The resin for printing was Anycubic Translucent Clear UV Resin (Anycubic, Shenzhen, China). The resin contained 38% iso-octylacrylate, 57.7% urethane acrylate, 4% of photoinitiator 2-hydroxy1-[4-(2-hydroxyethoxy)phenyl]−2-methylpropan-1-one) and 0.3% of pigment. Other technical specifications of the 3D printer and its translucent clear resin are listed in the electronic Supplementary Material (ESM, Table S1). The 3D-printed modular blocks were rinsed with industrial-grade ethanol employing INfusia SP7 syringe infusion pump (Frenesius Kabi, Bad Homburg vor der Höhe, Germany). The INfusia SP7 pump was also used for fluid injection to the fabricated modular fluidic platforms. Further post-processing for the modular blocks applied the clear gloss paint from Sapporo Ultimate Motorcycle Clear Gloss Spray Paint (PT. Warna Mikha Mitra Sejati, Bandung, Indonesia). The photos’ contact angle analysis was obtained using the ImageJ software (National Institutes of Health, Bethesda, MD, USA). Mixer simulations were performed by COMSOL Multiphysics 5.4 (COMSOL Inc., Columbus, MA, USA). The mesh strategy during the simulation was using a physical controlled mesh with a fine density option for laminar flow and transport diluted species model.

### 2.2. Design of Primary Modular Fluidic Platform

**Figure 1:**
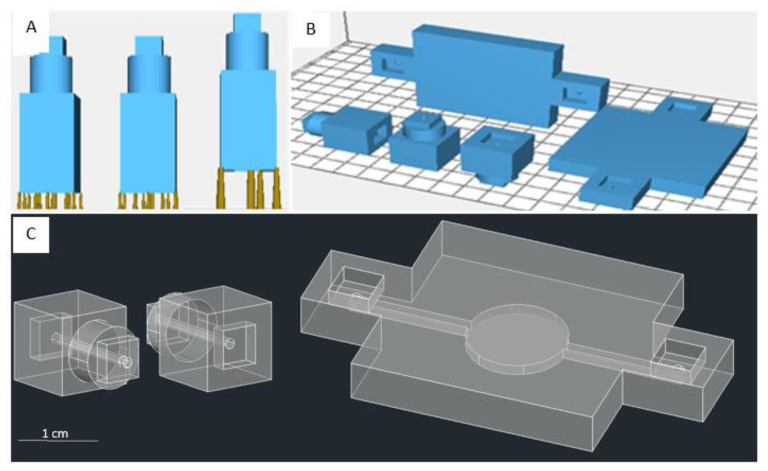
(**A**) The design of the inter-module connection. Each modular block has a pair of connecting structures on two ends. The CAD shows different support options: low, middle, and heavy supports. (**B**) Two printing directions, horizontal and vertical, with the reference of the printer platform. (**C**) The example of CAD drawings of connector modules (left and center) and chamber modules (right).

There were two main modular block designs for microfluidics purposes. The designs included a connecting module with a channel structure and another module with a chamber structure. Each modular block could be connected using a lock and key system. The lock was constructed by a crevice, imitating a plate with the key size (Figure 1A). Printing parameters, structural supports for printing (Figure 1B), printing orientation (Figure 1C), and post-processing techniques ensured the final product’s success. The standard dimension of the printed channels was adjusted to the diameter of the feeding tube. In this case, the diameter of the channels in the connector modules was 1.3 mm. We also designed two other orientations. The vertical orientation was printed perpendicular to the printer’s build plate, while the horizontal orientation was printed parallel to the printer’s build plate (Figure 1C). 

The fundamental connector block dimensions are shown in Figure 1C (left) and Figure 1C (center); they are 10 mm × 10 mm × 10 mm with a 2 mm × 5 mm × 5 mm lock and a cylinder with a radius of 8 mm and a length of 4 mm. The dimension of the fundamental chamber block in Figure 1C (right) is 30 mm × 30 mm × 5 mm, with the extension housing the lock structure having a dimension of 10 mm × 10 mm × 5 mm. The length of the inlet/outlet channels is 1 mm, connected to a 10 mm long channel with a 1.3 mm radius leading to the chamber. The diameter of the circle chamber is 6 mm. Other modules’ designs of connectors and chambers are shown in the Supplementary Materials (ESM, Figure S1).

### 2.3. Evaluation of Printing Parameters

In this study, we consider that several printing parameters are critical in printing a small structure (ESM, Figure S2). The parameters in CHITUBOX include the layer height, bottom layers, exposure time, bottom exposure time, light off delay, and bottom light off delay. The layer height controls the height of individual layers exposed to UV for the duration controlled by exposure time. Bottom layers set the number of initial layers exposed to a different UV set duration in bottom exposure time. Normally, bottom layers are exposed longer to UV to strengthen the structure’s connection to the build platform, allowing it to stick and not collapse onto the resin vat. Meanwhile, light off delay and bottom light off delay provides a delay after curing the respective individual layers.

The evaluated printing parameters are layer thickness and normal exposure time if using ANYCUBIC PhotonSlicer (ESM, Figure S2A), or layer height (LH) and exposure time (ET) if using CHITUBOX (ESM, Figure S2B). We used CHITUBOX software for printing a modular block close to or less than 1 mm in dimension because the ANYCUBIC PhotonSlicer could only set a minimum scale of two digits, while CHITUBOX was able to set the minimum scale of a single-digit micron. For example, ANYCUBIC PhotonSlicer would automatically change the layer thickness value to 1.03 mm when we inserted the value of 1.025 mm.

**Figure 2:**
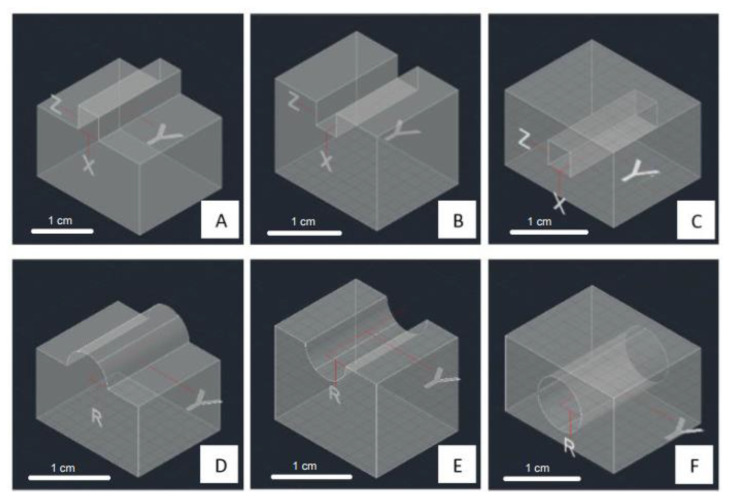
The design of single square-shaped (**A**) embossed, (**B**) debossed, and (**C**) hollow-style structures, and the design of single circle shape of (**D**) embossed, (**E**) debossed, and (**F**) hollow-style structures for basic printing structure evaluation.

**Figure 3:**
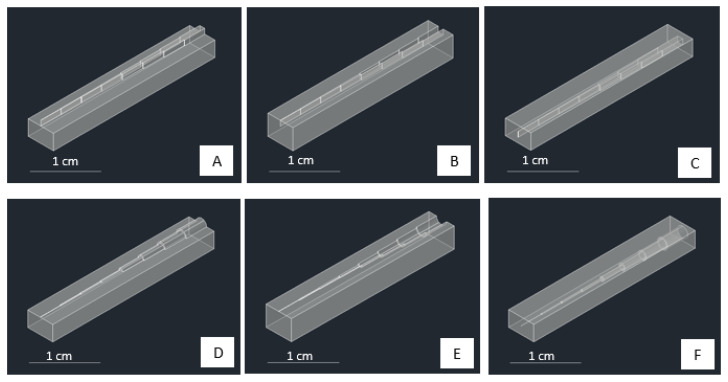
The designs of square-shaped (**A**) embossed, (**B**) debossed, and (**C**) hollow-style structures with decreasing size of the side’s square from end-left to end-right. The designs of circle-shaped (**D**) embossed, (**E**) debossed, and (**F**) hollow-style structures with decreasing size of circle’s radius from end-left to end-right.

To evaluate the two parameters mentioned previously, we designed two shapes of the structure, square and circle, and combined them with the three style structures, embossed, debossed, and hollow, as displayed in Figure 2. These two groups (shape and style) were related to the exposure time and printing orientation. The structures were designed to increase in size gradually. The square shapes have side dimensions of 50, 100, 150, 200, 400, 600, 800, and 1000 µm (Figure 3A–C). The circle shapes have a diameter of 100, 200, 300, 400, 800, 1200, 1600, and 2000 µm (Figure 3D–F). The distance between each changing size of side or diameter in the structure is 3.25 mm. We tested the exposure time from 5–10 s, as it was still in the range of the recommended exposure time following the printer manufacturer’s instruction (ESM, Table S1). These two structures were evaluated based on the dimension limit when the printing parameters created the structure.

The dimension limit in our measurement is the successfully fabricated structure from the design. The measurement were observed under the confocal microscope (Olympus, Tokyo, Japan), and measure the distance based on the scale from the microscope display. The deviation of the distance from the microscope scale to the original design was recorded as the standard error in the measurement.

### 2.4. Approach for Submillimeter-Scale Chamber Module

**Figure 4:**
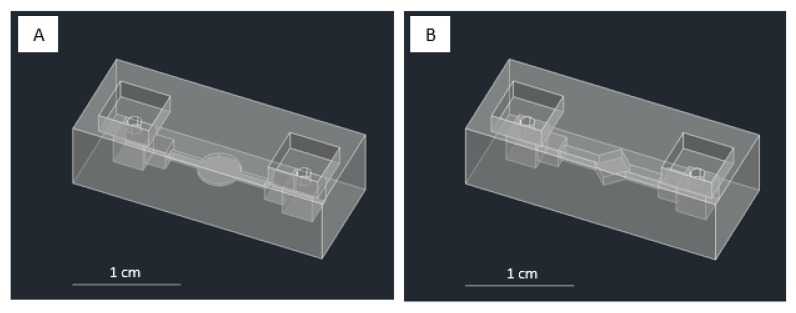
CAD designs of the chamber modules: (**A**) gradual circle chamber and (**B**) gradual diamond chamber.

For the chamber module, we implemented two designs: flat and gradual. The former implies that the chamber inside, including the channels, is lined up on the same surface and of the same size (Figure 1C (right)), while the latter refers to the significant change in the channels’ size leading up toward the chamber (Figure 4). The dimensions for the fundamental chamber blocks in Figure 4A,B are 25 mm × 10 mm × 7 mm with an inlet of 1 mm. The gradual chambers’ dimensions are 3 mm × 3 mm × 3 mm, 2 mm × 2 mm × 2 mm, and 4 mm × 1 mm × Z mm, respectively, leading into the circle chamber (Figure 4A) of 4 mm diameter and diamond chamber (Figure 4B) with a diagonal length of 4 mm. Z mm is the dimension of channel height that we evaluated and targeted to reach a submillimeter scale.

## 3. Results and Discussion

### 3.1. Basic Optimization Using the Test Model Setting

Our preliminary fabrication trials resulted in some common failures due to miscalibration, incorrect printing orientation, misplacement of printing supports, and imprecise printing parameters. The first important finding was related to miscalibration. In a bottom-up stereolithography printer, calibration is performed by setting the starting z-coordinate for the build plate and ensuring that the build plate is lying flat on top of the resin vat at an optimal distance. Misaligning the build plate leads to the fabrication success on only one side (left or right) of the printed area, even though identical elements are printed on both sides of the build plate. It is a reminder for meticulous adjustment, as the target structure would be in a millimeter or submillimeter scale; the scale uncommonly used on the low-cost 3D printer. Setting the starting z-distance could be crucial to the result; setting it too far makes some printed portions remain sticking on the resin vat, while setting it too close might induce the build plate to damage the UV light’s glass case (ESM, Figure S3A). Other consequences of miscalibration are generating defects in the fundamental block or housing of the fluidic channels themselves.

After z-coordinate calibration, the next challenge in fabricating the connector modules using default printing parameters (ESM, Figure S2B) is establishing the fluidic channel’s existence. The orientation of the fundamental block itself and the use of support plays a vital role. The channel has to be perpendicular to the build plate. Otherwise, the channels located inside the fundamental block would be flooded with uncured resin exposed to UV, blocking the channel entirely (ESM, Figure S3B). Setting the channel in a perpendicular orientation allows the uncured resin to flow out of the channels during the printing process due to gravity, avoiding unwanted UV exposure. However, printing the platform perpendicular to the build plate creates another problem, in which the cavity of the hole which serves as a lock is absent. The absence of the lock is the consequence of it being considered a ‘bottom layer’ exposed beyond the normal exposure time. Reducing the bottom exposure time is possible, although it might complicate the matter, as the bottom layer might not stick to the build plate.

To mitigate the problems above, adding the supports is essential for the fabrication process. Determining the size and placement of the supports could be quite an intricate process without the appropriate insight into how the system works. Removing unnecessary supports on the fundamental block might cause the block itself to collapse (ESM, Figure S3C). On the other hand, installing too few supports would allow the block to detach from its supports, failing the effort altogether. The supports act as an elevated platform from the build plate. The fundamental block needs to be printed on the supports rather than the build plate to avoid the above problems. With this strategy, the supports should be kept at a reasonable density, of which most slicing software offers automatic placing of supports.

**Figure 5:**
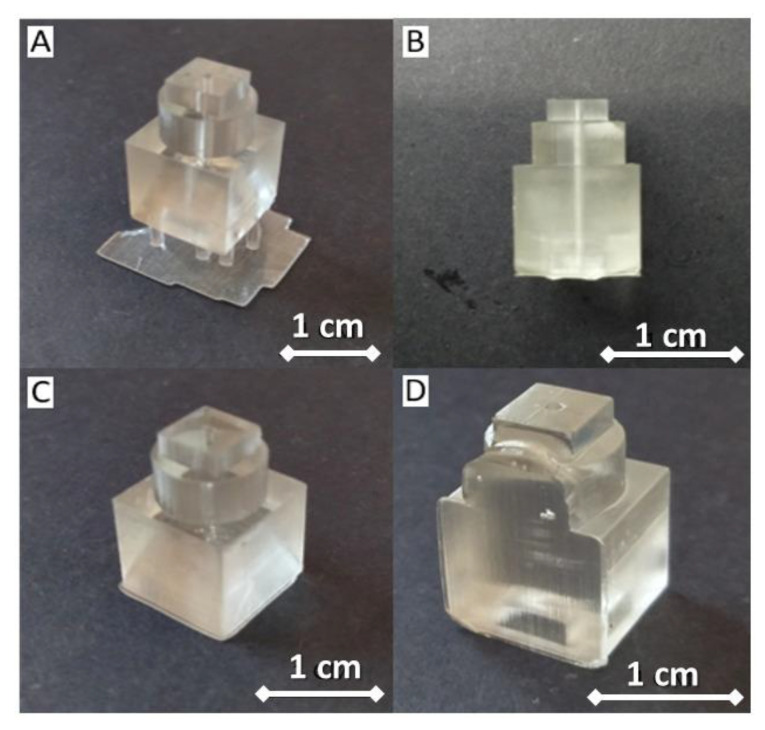
Printing parameters for connector module. (**A**) Vertical orientation with the added support structure. (**B**) Side view of connector module. The printed module with defects because of (**C**) lack of support structures and (**D**) using horizontal orientation design.

In contrast, printing the channel perpendicular to the build plate might also present a problem when dealing with observation chambers. The observation criteria need to have a clear surface, so the chambers are visible for observation. In bottom-up stereolithography, as layers are printed in a bottom-up direction, printing the chambers upright leaves the observing block quite translucent. To increase its transparency, the post-processing of the printed chamber module is noteworthy. Lastly, setting the suitable printing parameters shows its prominence in the fabricated modules’ results, as we noticed the failure in using the unfit setting of the printers (ESM, Figure S3D). For the fabrication of connector modules, we found that using the parameters from the default test model (ESM, Figure S2) added with a support structure and printed on vertical orientation gave successful printing results (Figure 5A,B), while non-supported design and horizontal printing orientation failed to fabricate the modules (Figure 5C,D). Therefore, we decided to reduce the layer height to the minimum setting (0.025 mm) of our connector set modules from the designs shown in Figure 1C (left and center) to minimize the risk of structural failures or channel collapsing if the build plate was drawn up (Z-direction) longer. The results are shown in the Supplementary Information (ESM, Figure S4), where we successfully printed six types of connector modules, from simple structures to complex structures.

### 3.2. Dimension Limit Investigation from Simple Structures

**Figure 6:**
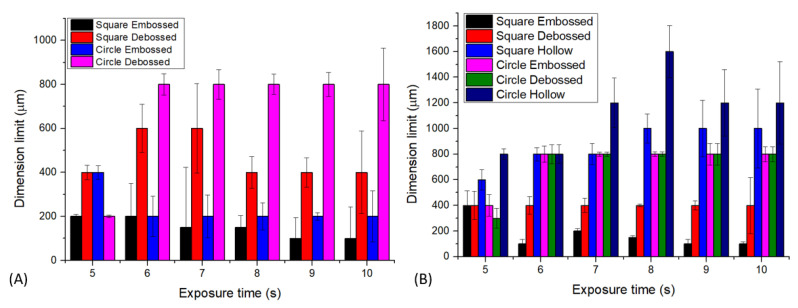
Dimension limit evaluation of different shapes and structures for (**A**) horizontal and (**B**) vertical printing orientations.

In understanding the dimension limit of successfully fabricated structure from the 3D printer machine, we consider checking the simple structure and starting from the limit or near the limit of the machine’s capabilities (ESM, Table S1). Figure 6 shows the summary of our findings. In the case of printing with horizontal orientation, we were unable to fabricate the hollow structures of both the square and circle shapes. A hollow structure is critical for creating a flow channel or a fluidic chamber for developing a fluidic platform. The dimensional limit of each case is defined as the smallest point or dimensional level at which the structure is fully constructed. Figure 6A,B represent that the smallest dimension formed in a square-embossed structure is 100 µm with an exposure time of 10 s, horizontal print orientation, and exposure time of 6, 9, and 10 s, vertical print orientation. For a square-debossed structure, the smallest dimension formed is 400 µm in the horizontal orientation with an exposure time of 5 and 8 s, in the vertical print orientation with an exposure time of 5–10 s. For the square-hollow structure, the structure is only formed in a vertical orientation with the smallest dimension up to 600 µm at the exposure time of 5 s.

Furthermore, Figure 6A,B also showed that circle-embossed shapes could be formed up to 200 µm with the horizontal print orientation and exposure time of 6–10 s. For circle-debossed, the smallest dimension formed is 300 µm with a vertical orientation and an exposure time of 5 s. For the circle-hollow shape, the smallest dimension formed is 800 µm with a vertical print orientation and exposure times of 5 and 6 s. We considered that the horizontal approach was not suitable for fabricating a hollow structure, contrasted with using a vertical orientation, where a minimum dimension can be printed up to 600 µm (vertical, square, hollow) and 800 µm (vertical, circle, hollow), presumably due to the uncured resin that remained in the channel. In the open-area printing case (embossed and debossed styles), we can see from Figure 6 that all structures could be printed up to a submillimeter scale. This condition is different from hollow structures because printing in an open environment design allows the uncured resin to be released or removed from the printing block during the printing process. Table 1 summarizes the best condition for obtaining the desired shape and style. Further evaluation of different structures may find the different optimum conditions that are also interested in further exploration.

### 3.3. Post-Processing and Printed Module Evaluation

**Figure 7:**
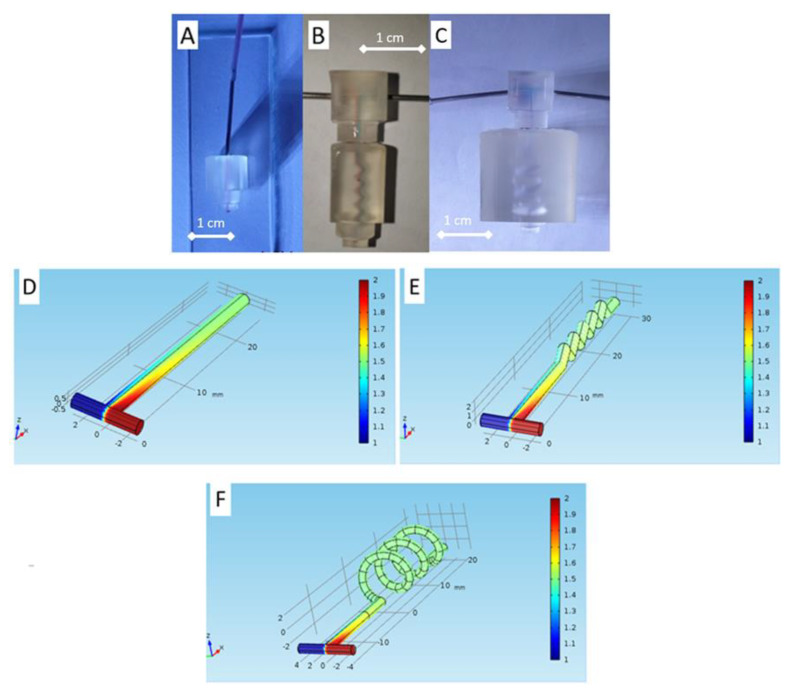
Flow observation on modular blocks. (**A**) Straight connector module, (**B**) T-channel with zig-zag connector module, and (**C**) T channel with spiral connector module. Simulation study for functional mixer connector using plasma blood sample (concentrations 1 and 2 mol/m^3^) for designed T channel (**D**) without mixer (**E**) with zigzag mixer, and (**F**) with spiral mixer.

After printing, we used simple post-processing to increase the printed modules’ transparency by adding a clear gloss on the platform’s surface (ESM, Figure S5). In addition, when creating channels on a millimeter or smaller scale, uncured resin tends to stay inside the channels due to capillary action. Without immediate treatment, the uncured resin could harden inside the channel due to ambient light, blocking the channel completely. Proper countermeasures, such as avoiding ambient light and circulating ethanol inside the chambers, settle this problem [31,34]. Fabricated block functions were evaluated by injecting colored water utilizing syringe pumps to ensure fluid flow within the channels/chambers and the integrity of connections between modules. During the experiment, connector modules were injected with colored water at a constant flow rate of 5 mL/h, and no leakage was observed (Figure 7A–C).

Furthermore, we observed by a simulation study that the proposed structures are potentially applied for plasma blood mixer devices. As depicted in Figure 7D–F, the uniformity of the concentration in the outlet can be achieved in T-channel, zigzag and spiral mixer devices. From the simulation results, the spiral mixer was able to mix the plasma blood sample immediately in the spiral structure, while for the T channel and zigzag mixer, the uniformity could be achieved in the outlet. The explanation for these phenomena could be that the spiral structure mixes the sample in a 3D flow direction, while the T channel and zig zag mixer flow in the 1D and 2D directions, respectively. 

The similar findings were obtained for the simulated connector–mixer microfluidics for liquid samples using phosphate buffer saline (PBS) and urine. The spiral mixer achieves a better uniformity of the mixed samples, compared to the T or zigzag structures as depicted in Figure S8.

**Figure 8:**
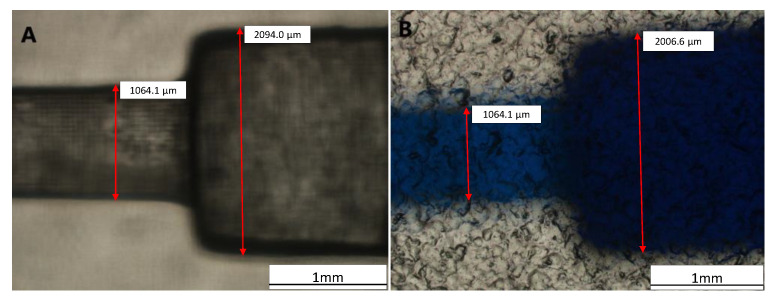
Measurement of the channel width in (**A**) without fluid and (**B**) with fluid by a confocal microscope.

The blocks had hydrophilic characteristics when a simple droplet test was conducted to determine the modules’ surface characteristics (ESM, Figure S6, Table S2). Therefore, the fluidic modules might serve as an alternative for oil-in-water droplet tests and other tests that require a hydrophilic medium [35]. Next, we tested two printing orientations and evaluated whether the surface hydrophilicity would change upon receiving a longer exposure time. From the results, we observed no distinct surface hydrophobicity change, and the surface remained hydrophilic, even at the maximum exposure time recommended by the 3D printer’s manufacturer (ESM, Figure S6, Tables S1 and S2). Additional observation through a confocal microscope was conducted to measure the dimensions of the printed module (Figure 8). Based on the direct measurement in Figure 8A, the bigger channel’s width was 2094.0 µm, and the smaller channel’s width was 1064.1 µm. Compared to the original design with 2000 µm of the channel width, there was a slight deviation of 94 µm or about 4.701% error in dimension. However, the direct measurement is prone to human error, as the object of interest has thick edges and is not perfectly transparent.

### 3.4. Chamber Modules and Strategy to Approach a Submillimeter Scale Structure

**Figure 9:**
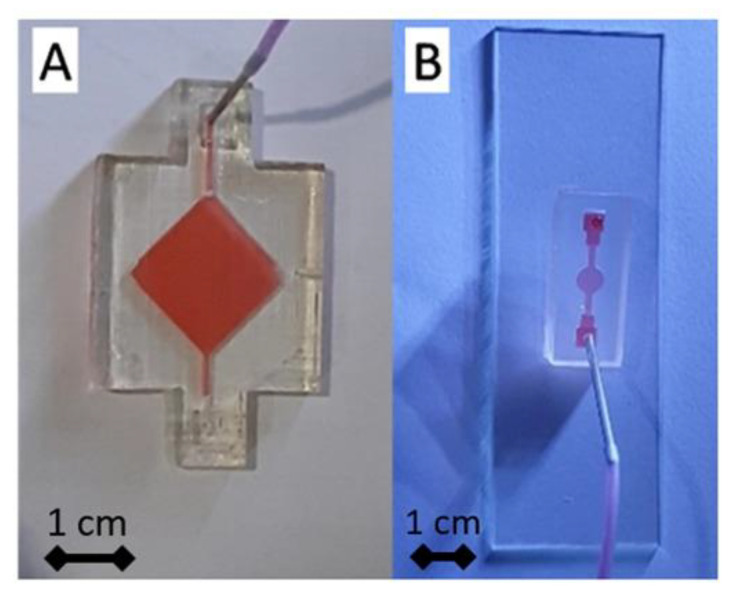
Flow observation on modular blocks. (**A**) Simple diamond chamber module, (**B**) Gradual circle chamber module.

Our designs focus on the chamber’s design as our targeted structure reaches the submillimeter scale. First, we used the test model parameters (ESM, Figure S2) to fabricate the design. However, our testing using the parameter from the printer’s default setting with a vertical orientation and support structure failed to fabricate structures with dimensions less than 1 mm using the designs of Figure 1C (right) and Figure S1F. In our initial design, on a larger scale, such as 1.3 mm, we successfully printed the chamber using a vertical orientation and added support (Figure 9A). Additionally, although we already evaluated the optimum printing condition as shown in Table 1, we realized that we needed to facilitate the different scales of the feeding tube to the fluidic channel/chamber scale in a hollow-like structure, where it could go down to a ten times smaller scale. Due to these conditions, we also designed a unique approach for reaching a submillimeter dimension of the channel and chamber height, using a gradual chamber design (Figure 4A,B differs only on the central chamber’s design), printing orientations, and modifying the printing parameters.

In the first evaluation, we noticed that reducing the exposure time from 8 s to 6 s could print the submillimeter dimensions using vertical and horizontal orientation without added support. We also discover that gradually diminishing the channel and chamber height allows the chamber to be connected in a shorter distance, approaching the structure from a millimeter scale (inlet-outlet area) to a submillimeter scale (narrow channels and chamber area) than by directly introducing a large gap (millimeter to submillimeter). For example, the structure collapsed if we designed a 5 mm inlet–outlet structure and a 0.9 mm channel–chamber structure. On the contrary, a gradual decrease in the dimension of 5 mm–3 mm–0.9 mm could be fabricated. Figure S7 shows the optimal printing parameters and the printing orientations we tested. We could fabricate chamber modules using horizontal orientation with the smallest dimension of 0.85 mm using these two strategies. However, better results were obtained when using a vertical orientation; a minimum structure of 0.55 mm could be created using a gradual structure of 5 mm–3 mm–0.55 mm (Figure 9B). From these results, we noticed that changing the printing orientation into vertical helped in defining narrow channel/chamber due to more reliable and precise printing from the 3D printer’s XY DPI specification (ESM, Table S1), rather than relying on z-direction movement (layer resolution) as in the case of printing using the horizontal orientation.

We realized that there is room for improvement from our current results, considering several non-optimized parameters, which may improve the dimension limit of the structure. Table 2 compares our work with the works from other groups utilizing 3D printing for a wide range of applications, such as drug dissolution assay [36], probes fabrication [34], or cell processing [37]. Several groups conducted evaluations of 3D printing for microfluidics fabrication, for example, testing the mail-order service for fabricating the microfluidics. Rogers et al. (2015) tested the creation of microfluidics channels with membrane-based valves integration, and Shallan et al. (2014) used 3D printing for fabricating micromixer, gradient generator, or droplet extraction, although the technical details on printing parameters were not mentioned [38,39]. Inverted microscope and scanning electron microscopy were used for the smallest printed dimension measurement [38,40,41,42,43].

Table 2 demonstrates the low investment cost of our study with about 20-fold cuts-off in the estimated unit price compared to the other reported works. This is also supported by the low-priced resin consumed in our 3D system. It is also noticeable that despite our dimension limit not being the smallest, this feature does not hinder the mixing performance, as shown in the simulation results. Even to compare with the 3D Systems Viper SL system, which is 20 times more expensive than our system, the dimension limit is merely 50 µm different. To achieve a smaller design, further adjusting the printing parameters is necessary for the resin polymer to properly 3D fabricate hollow structures and prevent the collapse of the hollow inner walls.

For a comparative study, we present the related literature in a comprehensive list in Table 3. From our literature studies, the resolution achieved by 3D printing technology that may not be as small as the softlithography. Nevertheless, the functional application of microfluidics, such as mixer, interconnecting channel, particle sorter, micropump and biosensing flow cell, have potential for the scale-up fabrication using 3D printing and cut the fabrication cost significantly.

From our study, it is shown that the low-cost 3D printer lends itself for a much bigger chance for research or small-scale production and is also reachable for the general user. Furthermore, we believe that the current works will impact the broader low-cost development of microfluidics modules for various purposes, such as their integration with the sensor as a flow cell or as an observation chamber, for instance, for in situ grown of biofilms [42], microluidic vascular channels [45], or sensor integration [46]. As for developing the microfluidics platform for the sample observation chamber, further testing and evaluation of compatible resins with higher transparency is of interest for future works.

## 4. Conclusions

We demonstrated a method to fabricate novel lock-and-key mixer modules and integrated chamber using a low-cost commercial 3D printer by adjusting the printing parameters, reconfiguring printing orientation, localizing printing supports, and adding post-processing techniques for modular blocks to give a clearer view of the surface, which is crucial for observation. The resolution down to 550 µm can be achieved and feasible for functional application microfluidics, such as a modular connector and mixer. Our proposed method provides a low-cost and straightforward alternative to conventional and costly monolithic microfluidics devices, which costs less than USD 400 for the equipment and USD 50 cents for each printed device. The fabricated microfluidics devices shows a potential application for biological sample preparation, such as plasma blood mixers, especially using the spiral channel, based on the simulation study that mixes different plasma concentrations of 1 and 2 mol/m^3^. Furthermore, our proposed methods can be translated to other low-cost commercial 3D printers with higher resolution, providing an alternative to small-scale production for other researchers toward future microfluidics platform developments.

## Figures and Tables

**Table 1:** Optimum printing setting with a minimum dimension.

Desired Structure	Optimum Setting
Square embossed	Vertical orientation; 6, 9, or 10 s exposure time
Circle embossed	Horizontal orientation; 6–10 s exposure time
Square debossed	Vertical orientation; 5–10 s exposure time
Circle debossed	Horizontal orientation; 5 s exposure time
Square hollow	Vertical orientation; 5 s exposure time
Circle hollow	Vertical orientation; 5–6 s exposure time

**Table 2:** The feature comparison of our work with previously published research in 3D-printed microfluidics.

No.	System	Printing Type	Pixel Resolution	Smallest Printed Dimension	Unit Price (Current Estimation)	Resin Price	Ref.
1	Miicraft	DLP-SLA	30–78 µm	250 µm	USD 8500–USD 12,500	USD 510/ 1 kg	[39]
2	Asiga Pico Plus 27	DLP-SLA	27 µm	150 µm	>USD 10,000	USD 175/1000 mL	[34]
3	Asiga Max-X27 UV	DLP-SLA	27 µm	120 µm	>USD 10,000	USD 175/1000 mL	[36]
4	3D Systems Viper SL system	DLP-SLA	75 µm	500 µm	>USD 10,000	-	[40]
5	Stratasys Objet Eden 350V	Polyjet	16 µm	100 µm	>USD 10,000	-	[37]
6	B9 Creator 3D printer	DLP-SLA	15 µm	250 µm	>USD 10,000	USD 299/1 kg	[38]
7	Anycubic Photon S	DLP-SLA	47 µm	550 µm	<USD 400	USD 25/500 mL	Our work

**Table 3:** Comparatve studies in literatures that presented related microfluidics using connector and mixer.

No.	Microfluidics System	Fabrication Method	Technical Remarks	Smallest Dimension	Potential Application	Ref.
1	USC-shaped fluid router and microfiller	3D printing	Vat photopolymerization	10 µm gap (microfiller)	Particles sorter	[32]
2	Centifugal microfluidic	CNC micromilling	Integrated to colorimetric	80 μm	Gas diffusion in analyte	[43]
3	Biosensing flowcell	Cyclic olefin copolymer (COC)	Integrated electrochemical	300 μm	Cell culture	[30]
4	Spiral microfluidics	Lithography	Simple particle size separation	75 μm	Circulating tumor cells (CTC) sorter	[41]
5	Interconnecting channel scaffolds	Material Extrusion 3D printers (mold) and PDMS casting	Single-extrusion scaffolds	100 μm	Mixer iquid sample and droplet generator	[44]
6	Microscale impeller pump for recirculating fluid flow	3D printing	magnetically-driven impeller pump system	500 μm	Organ-on-chip and mircoreactor	[7]
7	Modular key–lock and mixer connector	3D printing	Fully portable integrated 3D spiral mixer	550 µm	Biofluid mixer, such as urine and blood	This work

## Supplementary Materials

The following are available online at https://www.mdpi.com/article/10.3390/mi13081197/s1, Figure S1: Designs of other modules, Figure S2: One of the test model structure from ANYCUBIC PhotonSlicer and CHITUBOX, Figure S3: Fabrication failures, Figure S4: Fabrication result of connector modules, Figure S5: Surface condition of fluidic module, Figure S6: Evaluation of contact angle from three spots using sofware ImageJ, Figure S7: Optimal printing conditions, Figure S8: The simulated performance of integrated connector and mixer for biochemical samples for different structures, Table S1: Anycubic Photon S Technical Specifications, Table S2: Contact angle evaluation.
