# Supplementary material for: Versatile and Low-Cost Fabrication of Modular Lock-and-Key Microfluidics for Integrated Connector Mixer Using a Stereolithography 3D Printing
Source: Micromachines (Basel). 2022 Jul 28;13(8):1197. doi: 10.3390/mi13081197 (PMC9413493; doi:10.3390/mi13081197)
Supplement: Supplementary file 1 [file micromachines-13-01197-s001.zip › Supplementary Material.pdf]

Supplementary Material

Versatile and low-cost fabrication of modular microfluidics using a stereolithography 3D printing

Isa Anshori[1,2,*], Vincent Lukito[1], Rafita Adhawiyah[1], Delpita Putri[1], Suksmandhira Harimurti[1], Tati Latifah Erawati Rajab[1], Arfat Pradana[2], Mohammad Akbar[3], Mas Rizky Syamsunarno[4], Murni Handayani[5], Agnes Purwidyantri[5,6], Briliant Adhi Prabowo[5,6,*]

[1]Biomedical Engineering Department, School of Electrical Engineering and Informatics, Bandung Institute of Technology, Bandung, Indonesia
[2]Research Center for Nanosciences and Nanotechnology (RCNN), Bandung Institute of Technology, Bandung, Indonesia
[3]Department of Cardiology and Vascular Medicine, Faculty of Medicine, Universitas Padjadjaran, Dr. Hasan Sadikin General Hospital, Bandung, Indonesia
[4]Department of Biomedical Sciences, Faculty of Medicine, Universitas Padjadjaran
[5]National Research and Innovation Agency (BRIN), Indonesia
[6]International Iberian Nanotechnology Laboratory (INL), Braga, Portugal

[*]Corresponding authors: isaa@staff.stei.itb.ac.id; briliant.prabowo@inl.int

Various complex designs of the connector were tested. The dimension of the fundamental blocks shown in Figure S1A is 10×10×10 mm with a 2×5×5 mm lock and a cylinder with a radius of 8 mm and a length of 4 mm. The multi-inlet multi-outlet dimension (Figure S1A) is 20×15×15 mm, with the lock adjusted into 2×7×7 mm to contain two channels inside it. The cylinder size is also adjusted into having a 12 mm diameter and 4 mm length. Figure S1B also shows the design of two inlets-one outlet for connecting two liquids, or vice versa, splitting one liquid into two liquids. Meanwhile, to accommodate a 3D spiral channel with 15 mm long and a constant radius of 3 mm, the dimension of the fundamental block in Figure S1C is modified to 20×20×20 mm.

In Figure S1D, we implemented a zig-zag pattern with a 2 mm long starting channel, followed by a 45° zig-zag channel with each branch having a length of 2 mm. Then, the zig-zag pattern is connected to the outlet with a 2.86 mm long channel. The dimension of this particular design is also adjusted to 15×10×10 mm. For the multi-inlet single-outlet design (Figure S1E), the fundamental block's dimension is 15×15×15 mm, with each inlet adjusted into a 45° arc, except for the vertical channel, meeting at the center of the block. Lastly, the dimension for the fundamental chamber blocks in Figure S1F is 30×30×5 mm, with the extension housing the lock structure having a dimension of 10×10×5 mm. The length of the inlet/outlet channels is 1 mm, connected to a 10 mm long channel with a 1.3 mm radius leading to the chamber. The length of the diagonal of the diamond is 12 mm long.

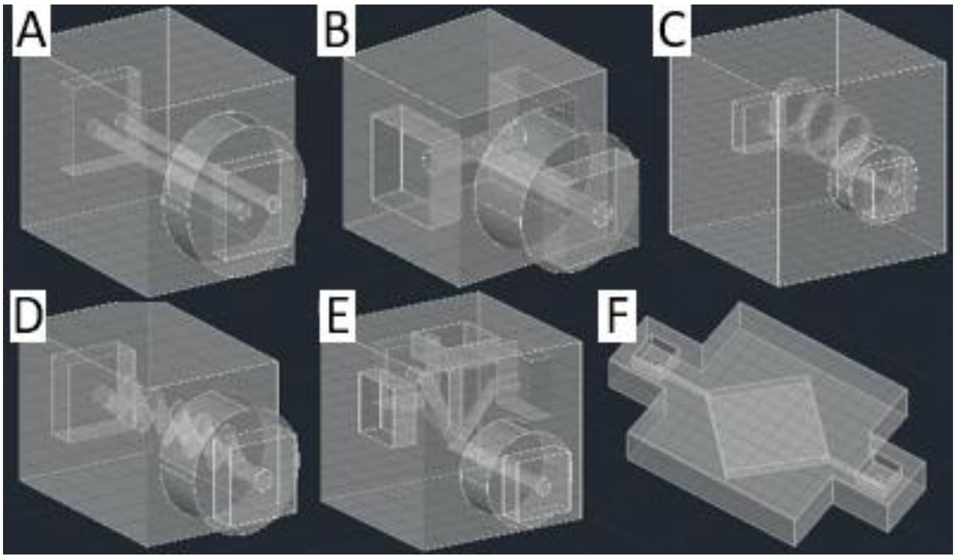

Figure S1. Designs of other modules

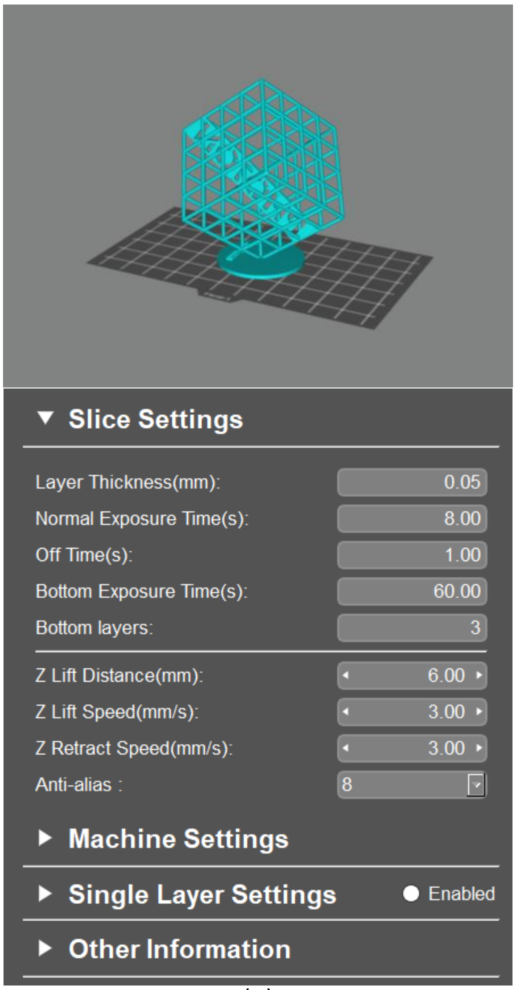

(A)

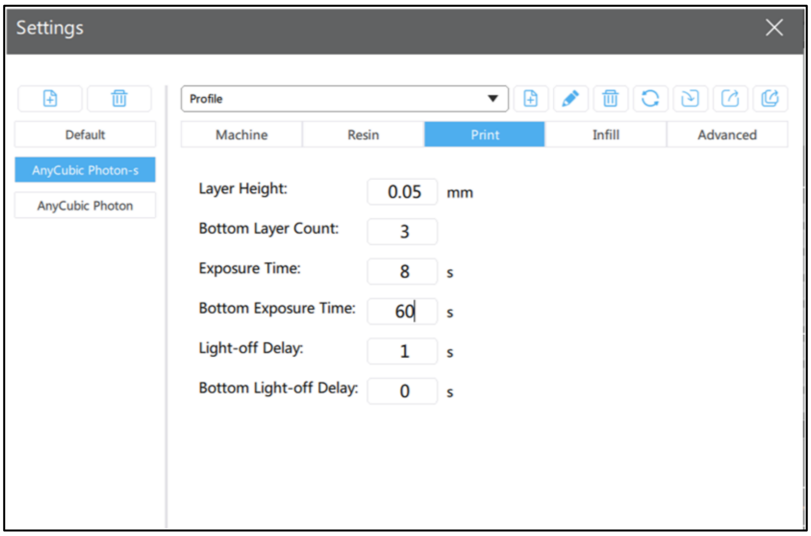

(B)

Figure S2. One of the test model structure from ANYCUBIC PhotonSlicer with (A) its printing parameters and (B) printing parameters opened in CHITUBOX.

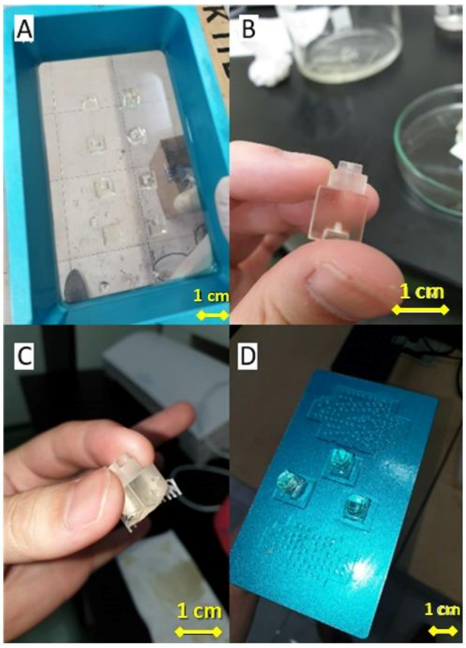

Figure S3. Fabrication failures. (A) Fabricated module failures due to miscalibration. The modules were stuck and partially cured on the resin vat instead of the build platform. (B) Incorrect orientation. Printing the channels in a parallel orientation allows for no uncured resin to escape which will block the entirety of the channel. (C) Misplacement of supports caused the block to collapse. (D) Incorrect printing parameters caused overall fabrication failure for certain modules.

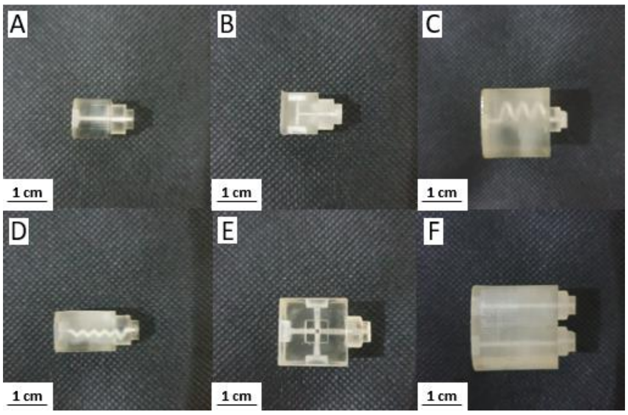

Figure S4. Fabrication result of connector modules with (a) straight channel design, (b) T channel design, (c) spiral channel design, (d) zig-zag channel design, (e) multi-inlet single outlet design, and (f) multi-inlet multi-outlet design.

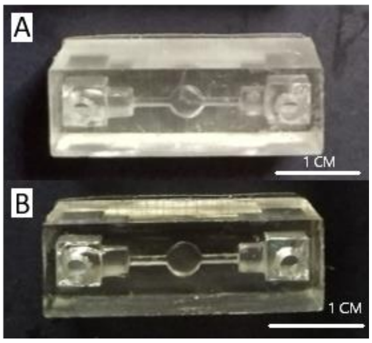

Figure S5. Surface condition of the microfluidic module (A) before and (B) after applying clear gloss paint.

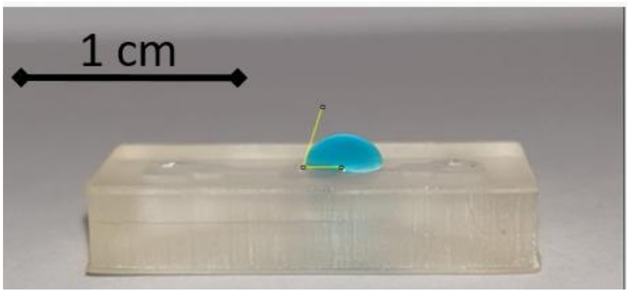

Figure S6. Evaluation of contact angle from three spots using ImageJ software.

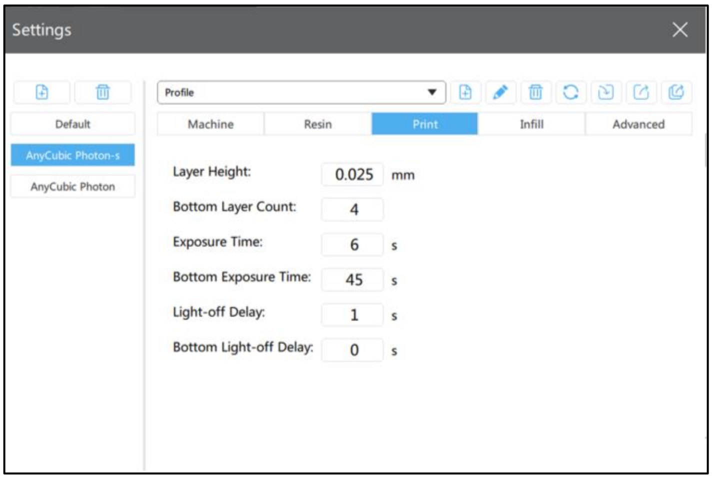

(A)

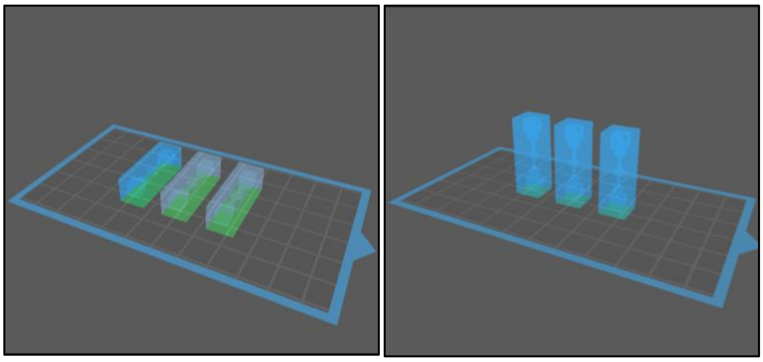

(B)

Figure S7. Optimal printing conditions. (A) Optimal printing parameters for chamber module. (b) Orientation evaluation and its support placement for vertical printing on chamber modules, while horizontal printing has no support.

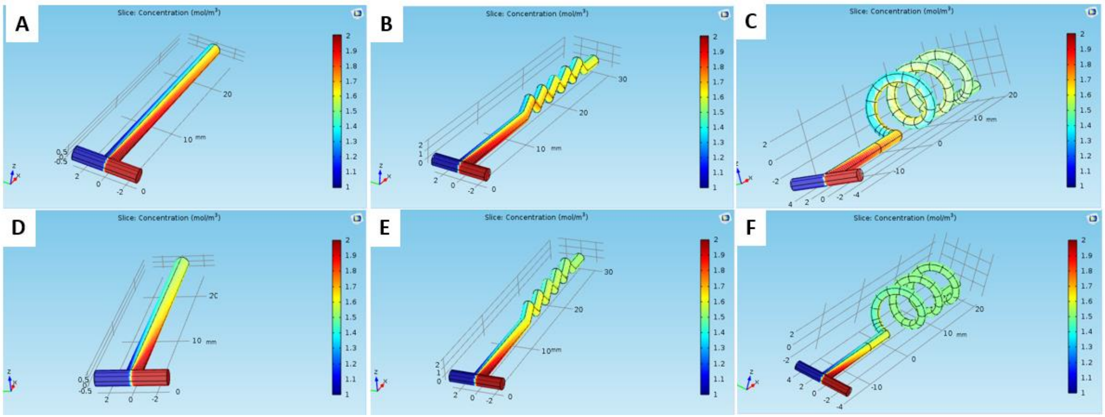

Figure S8. The simulated performance of integrated connector and mixer for biochemical samples for different structures for (A and D) T connector, (B and E) T connector and zigzag mixer, (C and F) T connector and spiral mixer. The simulated sample for (A-C) were using PBS samples and (D-F) were using urine, with the mixing concentrations of 1 and 2 mol/m^3.

Table S1. Anycubic Photon S Technical Specifications

No.	Specification	Value/Information
1	Printing Technology	LCD-based SLA 3D Printer
2	Light-source	UV integrated light (wavelength = 405 nm)
3	XY DPI	47 µm (2560 × 1440)
4	Layer resolution	25-100 µm
5	Printing material	405 nm photosensitive resin
6	Printing volume	115 mm × 65 mm × 165 mm (4.52″ × 2.56″ × 6.1″)
7	Range of exposure time	5-15 s for translucent clear resin

Table S2. Water contact angle evaluation

Printing orientation	Exposure time (s)	1st spot contact angle (°)	2nd spot contact angle (°)	3rd spot contact angle (°)	Average contact angle (°)
Parallel	5	57.54	63.05	64.29	61.63
	6	55.03	61.98	61.25	59.42
	7	60.75	66.37	78.09	68.40
	8	53.65	62.46	61.01	59.03
	9	61.93	68.22	64.72	64.95
	10	60.01	64.35	52.47	58.94
	11	59.40	64.06	56.60	60.01
	12	56.42	58.11	57.53	57.35
	13	59.39	54.87	58.16	57.47
	14	56.93	59.14	65.13	60.40
	15	59.24	63.55	62.95	61.91
Vertical	5v	55.33	74.36	69.40	66.36
	6v	55.36	76.81	74.20	68.79
	7v	63.46	66.75	58.70	62.96
	8v	67.62	70.07	69.83	69.17
	9v	69.51	65.10	67.48	67.36
	10v	66.39	73.68	69.08	69.71
	11v	45.89	60.53	62.97	56.46
	12v	52.39	62.44	63.34	59.39
	13v	61.74	56.26	60.91	59.63
	14v	64.15	60.04	58.67	60.95
	15v	59.78	67.92	61.65	63.11
